# A comprehensive evaluation of the accuracy of cervical pre-cancer detection methods in a high-risk area in East Congo

**DOI:** 10.1038/sj.bjc.6605594

**Published:** 2010-03-02

**Authors:** S Hovland, M Arbyn, A K Lie, W Ryd, B Borge, E J Berle, H Skomedal, T M Kadima, L Kyembwa, E M Billay, D Mukwege, R B Chirimwami, T M Mvula, P J Snijders, C J L M Meijer, F Karlsen

**Affiliations:** 1 NorChip AS, Industriveien 8, Klokkarstua 3490, Norway; 2 Unit of Cancer Epidemiology, Scientific Institute of Public Health, Juliette Wytsmanstreet 14, Brussels B1050, Belgium; 3 Department of Pathology, The Norwegian Radiumhospital, Montebello, Oslo 0310, Norway; 4 Department of Cytology, Sahlgrenska University Hospital, Göteborg 413 45, Sweden; 5 Department of Gynecology, Ullevål University Hospital, Oslo 0407, Norway; 6 Centre Hospitalier CELPA, PO Box 266, Bukavu, DR Congo; 7 Panzi Hospital, PO Box 266, Bukavu, DR Congo; 8 General Hospital, PO Box 266, Bukavu, DR Congo; 9 Department of Pathology, Vrije Universiteit Medical Center, PO Box 7057, Amsterdam 1007 MB, the Netherlands

**Keywords:** CIN, PreTect HPV-Proofer, E6/E7 mRNA, HPV DNA, RLB, Africa

## Abstract

**Background:**

Given the high burden of cervical cancer in low-income settings, there is a need for a convenient and affordable method for detecting and treating pre-cancerous lesions.

**Methods:**

Samples for comparing the accuracy of cytology, virology and histology were collected. Identification of HPV E6/E7 mRNA was performed using PreTect HPV-Proofer. HPV DNA detection was performed by GP5+/6+ PCR, followed by reverse line blot (RLB) for typing.

**Results:**

A total of 343 women, aged 25–60 years, attending gynaecological polyclinics in DR Congo were included for sample enrolment. The test positivity rate was conventional and liquid-based cytology (LBC) at cutoff ASCUS+ of 6.9 and 6.6%, respectively; PreTect HPV-Proofer of 7.3% and consensus DNA PCR for 14 HR types of 18.5%. Sixteen cases of CIN2+ lesions were identified. Of these, conventional cytology identified 66.7% with a specificity of 96.2%, LBC identified 73.3% with a specificity of 96.9%, all at cutoff ASCUS+. HR-HPV DNA detected all CIN2+ cases with a specificity of 85.9%, whereas PreTect HPV-Proofer gave a sensitivity of 81.3% and a specificity of 96.6%.

**Conclusion:**

Both HPV detection assays showed a higher sensitivity for CIN2+ than did cytological methods. Detecting E6/E7 mRNA from only a subset of HR HPVs, as is the case with PreTect HPV-Proofer, resulted in a similar specificity to cytology and a significantly higher specificity than consensus HR HPV DNA (*P*<0.0001).

In African countries, the incidence of cervical cancer is high, particularly in Sub-Saharan Africa, where cervical cancer is the most common form of cancer among women (Parkin *et al*, 1999). The majority of these countries have not been able to introduce a cytological screening programme because of the lack of resources and a sufficiently well-developed infrastructure, besides the fact that cervical cancer screening unfortunately is one of the many competing health priorities in these countries.

The incidence of cervical cancer shows a dramatic difference between high-income and low-income countries. Up to 80% of women in low-income areas suffer from advanced disease without possibilities for effective treatment (Goldie *et al*, 2001). Many lives might be saved by screening for pre-cancerous lesions and treatment of detected pre-cancers with the appropriate technology.

Different methods for detecting cervical cancer exist: Traditional morphological methods such as cytology (conventional PAP or liquid based), naked eye visual inspection using acetic acid or lugol iodine of the uterine cervix and molecular methods for HPV detection (Arbyn *et al*, 2008).

The HPV DNA and mRNA analyses have been introduced on the basis of the evidence that persistent presence and activity of oncogenic types of HPV is a necessary prerequisite for the development of virtually all cervical cancers (Bosch *et al*, 2002; Bosch and de Sanjose, 2003; Schiffman *et al*, 2005b). In general, HPV infections clear spontaneously. However, continuous expression of the viral oncogenes E6 and E7 is believed to be a necessary factor in the progression towards cervical cancer (zur Hausen, 2002).

This study aimed at determining the test accuracy of colposcopy, conventional cytology and liquid-based cytology (LBC), in addition to HPV DNA- and mRNA-based assays, for detecting histologically confirmed cervical intraepithelial neoplasia grade 2 or worse (CIN2+) in a mixed population (consisting of both screening and obstetrical/gynaecological problems), to determine the most effective screening tool for pre-cancerous lesions in African countries. The study was set up in a high-risk urban area in the eastern part of the Democratic Republic of Congo, which has been affected by civil war and violence against women for more than a decade.

In low-income countries, screen and treat strategies, with complex triage schemes, are preferable, but specificity is then an indispensable criterion to avoid treating large proportions of the screened population. In this paper, such a strategy was developed. A high-risk population was chosen, to guarantee identification of cases of CIN2+. Sensitivity and specificity estimates can be considered as representative for an African female population consulting gynaecological services; however, positive predictive values are expected to be inflated because of the relatively high prevalence of CIN2+.

## Materials and methods

The study was approved by the ‘Regional Committees for Medical Research Ethics’ in Norway and was performed in accordance with the ethical standards set by the Declaration of Helsinki 1975, revised 2000. It was carried out in agreement with the ethical recommendations of the provincial health authorities in Bukavu because of the absence of a local ethical committee. All aspects of the study protocol was thoroughly discussed by Congolese physicians, co-authors of this paper, who were highly qualified to evaluate any ethical issues.

Women attending three gynaecological clinics in Bukavu, Democratic Republic of Congo, during November and December 2003 were recruited for the study.

**Figure 1:**
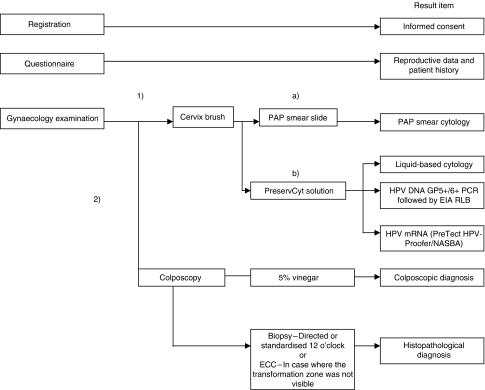
Examination and sampling procedure. The symbols (1) and (2) indicate the order of examinations. The symbols (a) and (b) indicate that the cervix brush was first used for PAP smear preparation before the brush was submerged into 20 ml PreservCyt solution. ECC, endocervical curettage.

A health worker informed all women about the study. A printed consent form translated into the local language was signed by the participants. Demographic data and information about reproductive health were obtained. Exclusion criteria were pregnancy, severe gynaecological bleeding, previous hysterectomy and age <25 or >60 years. The examination and sampling procedures are presented in Figure 1. Colposcopy diagnoses were verified by two physicians by joint examination and consensus. All women with CIN2 or worse lesions were offered appropriate treatment free of charge after the histology diagnoses were obtained and transferred to the physicians in charge of the follow-up of patients.

### Training of health professionals involved in sample collection

Gynaecological examinations were carried out by gynaecologists and general practitioners, all of whom received intensive training during a 2-week introduction course given by a Norwegian expert in low-resource gynaecology and gynaecological oncology.

### Collection and transport of samples

PAP smears, PreservCyt vials and histological material were stored at room temperature and shipped to Norway and Sweden for preparation and interpretation by well-experienced cytotechnicians and pathologists.

### Histology

Histological material (colposcopically directed or standardised biopsies at 12 o’clock position and/or endocervical curettage (ECC)) was obtained from all women for gold standard verification of the disease status, thereby detecting as many women with cervical disease as possible and also reducing verification bias.

Biopsy samples and ECC material were immediately transferred into PreservCyt vials. Histological examinations were carried out by an experienced pathologist (AKL) at the Norwegian Radium Hospital (Oslo) according to the WHO classification (Kurman *et al*, 1992). In 13 of the cases, more than one sample was taken from the same woman (one punch biopsy and one ECC or two punch biopsies). In three other cases, an additional cone biopsy was obtained to confirm the suspicion of high-grade disease on the basis of other test results. The score of the highest histology grade was considered in statistical analysis.

### Colposcopy

A colposcopy-directed biopsy sample was taken if any acetowhite lesion, mosaic, punctation, atypical vessels or other abnormal lesions were observed. Otherwise, a standardised biopsy sample at the 12 o’clock position was taken.

### Cytology

All conventional PAP smears were examined at the Department of Cytology at the Norwegian Radium Hospital by one experienced cytotechnician (BB) and positive smears were reviewed by a cytopathologist (BR). Slides for LBC were prepared and interpreted at the Sahlgrenska University Hospital (Gothenburg, Sweden) using a ThinPrep-3000 processor and an automated slide preparation unit, producing uniform thin-layer slides. All results were categorised according to the 2001 version of The Bethesda System (Solomon *et al*, 2002).

### DNA/RNA isolation from cytological cervical material

Nucleic acids (DNA/RNA) were isolated at NorChip AS (Klokkarstua, Norway) using the NucliSENS manual extraction kit (bioMerieux, Marcy l’Etoile, France) as previously described (Boom *et al*, 1990). DNA/RNA was isolated from 5 ml of the 20 ml PreservCyt vial. The 5 ml PreservCyt specimens were centrifuged for 10 min at 2500 r.p.m. and the pellets were washed with 70% ethanol before the addition of 900 *μ*l lysis buffer (NucliSENS, bioMerieux). The extracts were stored at −80°C.

### HPV RNA analysis by NASBA

For RNA detection, the RNA-based real-time nucleic acid sequence-based amplification assay (NASBA) was used, focusing on the reportedly most common oncogenic HPV types 16, 18, 31, 33, 35, 45, 51, 52 and 58 (Clifford *et al*, 2003a).

#### (i) HPV type 16, 18, 31, 33 and 45 (PreTect HPV-Proofer)

The HPV mRNA analysis was performed using the NASBA-based assay PreTect HPV-Proofer (NorChip AS) according to the manufacturer's instructions (a similar product is also marketed under the brand name NucliSENS EasyQ, bioMerieux).

To avoid false negatives due to degradation of mRNA, primers and probes against human U1A mRNA are included in the PreTect HPV-Proofer kit as a performance and integrity control.

#### (ii) HPV type 35, 51, 52 and 58

Presence of HPV 35, 51, 52 and 58 E6/E7 mRNA was analysed by NASBA using specific primers and molecular beacons for each HPV type. Artificial oligonucleotides corresponding to the viral mRNA were used as positive controls (NorChip AS). Negative controls consisted of Rnase-free water and were included in each run.

### HPV DNA analysis by GP5+/6+ PCR

The same nucleic acid extracts were used both for the NASBA assays and for HPV DNA detection by PCR, the latter using the consensus primer GP5+/6+ with an enzyme immunoassay readout as previously described (Jacobs *et al*, 1997; Snijders *et al*, 2005). EIA was performed using two oligoprobe mixtures A and B. Mixture A contained probes targeting the high-risk or putative high-risk HPV types 16, 18, 26, 30, 31, 33, 34, 35, 39, 45, 51, 52, 53, 56, 58, 59, 64, 66, 67, 68, 69, 73, 82 (variant MM4) and 82 (variant IS39). Mixture B targeted the low-risk HPV types 6, 11, 32, 40, 42, 43, 44, 54, 55, 57, 61, 70, 71, 72, 81, 83, 84, 85, 86, CP6108 and JC9710. Samples positive for either of the EIA mixtures were further typed by reverse line blot analysis (RLB). PCR products of the *β*-globin gene were included as DNA control.

### RLB of GP5+/6+ PCR products

Reverse line blot typing was performed using a previously described assay (van den Brule *et al*, 2002; Snijders *et al*, 2005) for detection of 39 HPV types individually (i.e., HPV 6, 11, 16, 18, 26, 30, 31, 33, 34, 35, 39, 40, 42, 43, 44, 45, 51, 52, 53, 54, 55, 56, 57, 58, 59, 61, 64, 66, 67, 68, 69, 70, 71, 72, 73, 81, 82/MM4, 82/IS39 and CP6108) and of six rare HPV types (i.e., HPV 32, 83, 84, 85, 86 and JC9710) as a pool.

GP5+/6+ PCR, EIA and RLB genotyping procedures were performed at the Department of Pathology, Vrije Universiteit Medical Center in Amsterdam, the Netherlands.

For the purpose of analysis in this study, we considered the following 14 HPV types as being of high risk: 16-18-31-33-35-39-45-51-52-56-58-59-66-68 (IARC, 2007). The other types were considered as low risk.

In general, all laboratory testing procedures were performed blindly without knowledge of other test results (except for the joint colposcopy examination).

### Statistical analysis

Test positivity rates and accuracy for underlying CIN2+ were computed for each of the different methods. McNemar's *χ*^2^-test was used to study associations between two categorical variables. Exact binomial confidence intervals (CIs) were computed where appropriate (Arbyn and Hovland, 2006).

## Results

A total of 343 women between 25 and 60 years of age (median: 37 years) were included in this study. The women came from different locations from the province of South Kivu (Democratic Republic of Congo).

**Figure 2:**
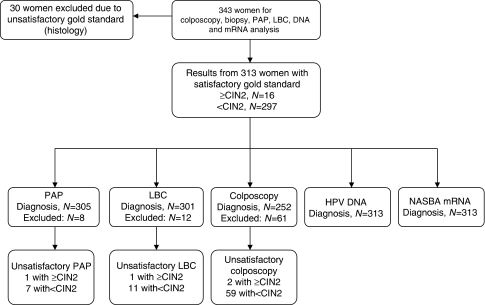
Overview of the material.

**Figure 3:**
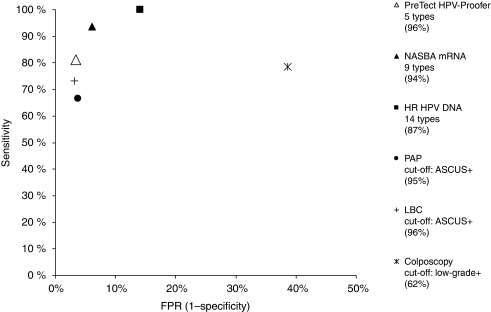
ROC diagram showing the accuracy (%) of all methods.

In Figure 2, some descriptive statistics of the participants are given. An overview of the different test characteristics of each assay is presented in Table 1, whereas in Figure 3, sensitivity values of each test are plotted as a function of the false positivity rate (1 specificity).

### Histology

Altogether, 16 out of 343 women had CIN2+ (eight cases with CIN3), which translates into a prevalence of 4.7% (95% CI: 2.7–7.5%). No cancers were diagnosed in the study, most probably because severe gynaecological bleeding was one of the exclusion criteria. Histology was unsatisfactory in 30 cases (8.7%), and these cases were left out of the overall statistical calculations, leaving a total of 313 cases. Three of the 16 CIN2 or worse lesions were identified in cone specimens, which were taken because of jointly positive cytology (HSIL) and nucleic acid assays (HR types) in spite of benign/low-grade histology in the punch biopsy.

Four of the 16 (25.0%) CIN2 or worse lesions were diagnosed in women from whom standardised punch biopsy samples were taken because of the absence of colposcopically suspicious areas.

### Colposcopy

In 72 of the 343 (21.0%) women, a colposcopic normal impression was observed, but colposcopy was regarded as unsatisfactory because the squamocolumnar junction was not visible.

In 40.9% of women, a positive colposcopic finding was noted, whereas in 6.3% a CIN2 or more serious lesion was suspected. Regarding the unsatisfactory colposcopic impressions, the histological outcomes were in 88.9% obtained by sampling with ECC, the results of which were benign in 79.7% of cases, low grade in 6.3%, high grade in 1.6% and unsatisfactory in 12.5%.

### Cytology

Nine PAP (2.6%) and 14 liquid-based smears (4.1%) were assessed as unsatisfactory. Almost 7% of satisfactory smears were considered abnormal (ASCUS+, Table 1): HSIL or worse was reported in 2.1% of conventional smears and in 2.4% of liquid-based smears.

### HPV DNA in cytological cervical material

The HPV DNA results obtained in the cytological material are presented in Tables 1 and 2. All samples that were negative by GP5+/6+ PCR tested positive by the *β*-globin DNA control. The PCR amplification followed by the EIA revealed the presence of HPV DNA in 104 out of the 343 women (30.3%). In all, 77 of the 104 HPV DNA-positive cases (74.0%) were positive by the EIA mixture A, which included all high-risk HPV types; 46 of 104 (44.2%) were positive for the EIA mixture B and 19 of 104 (18.3%) cases were positive for both the A and B mixtures.

When unsatisfactory histology samples (*n=*30) were excluded and the subsequent genotyping data were limited to the 14 HR types or the five HR types targeted by the PreTect HPV-Proofer, the positivity rate was reduced to 58 (18.5%) or 28 cases (8.9%), respectively.

Out of 58 HR HPV DNA-positive samples, the most frequent type was HPV 35, followed by HPV 45. HPV 16 took the third rank. A single infection was observed in 62.1% of HPV DNA-positive women, whereas 24.1% had a double infection and 13.8% carried three or more types.

When comparing DNA positivity (14 HR types) with the different histological diagnoses (*n*313), 13.9% were found to be DNA positive in histologically normal cases, 15.8% in HPV/CIN1 lesions and 100% in CIN2 or more serious lesions. Twelve of the 16 (75.0%) CIN2+ cases tested positive for DNA of HPV types 16, 18, 33 and/or 45. Eight of the CIN2+ cases were diagnosed as CIN3 and all were DNA positive, which gave 100% sensitivity and 83.6% in specificity (accuracy of 84.0%).

The most ideal combinations of HPV types giving maximum sensitivity and specificity for detection of CIN2+ by using cocktails targeting increasing numbers of HPV types are shown in Table 2. Ranking was carried out by increasing each step with the type that yielded the highest increase in sensitivity and when the increase in sensitivity was the same for two or more types, the one that resulted in the lowest loss in specificity was chosen. When the increase in sensitivity and loss in specificity were equal for two or more types, the type ranked highest in the list of most prevalent HPV types in cancer cases as established by Munoz *et al* (2004) was chosen.

When we used the same criteria as described above but confined the analysis to the five types included in the PreTect HPV-Proofer assay, thereby allowing a direct type-to-type comparison between the DNA and the mRNA assay, we found a maximum sensitivity of 75.0% with a specificity of 94.6%.

Sensitivity of HR-HPV DNA detection (i.e., 14 types) for CIN2+ (100.0% 95% CI:82.9–100.0%) was significantly higher than that of both cytology methods, even at cutoff ASCUS+ (PAP: 66.7% 95% CI:38.4–88.2% *P* for McNemar's *χ*^2^=0.014, LBC: 73.3% 95% CI:44.9–92.2% *P* for McNemar's *χ*^2^=0.025). Furthermore, the specificity of the HR-HPV DNA assay was significantly lower than both cytology methods at cutoff ASCUS+ for outcome CIN2+ (*P* for McNemar's *χ*^2^ lower <0.0001).

### HPV mRNA in cytological cervical material

The HPV mRNA results obtained in the cytological material are presented in Tables 1, 3A and 3B.

All samples tested positive for the RNA control U1A. PreTect HPV-Proofer yielded a positive result in 26 cases (7.6%). The most frequently detected HPV mRNA type was 45, followed by HPV 18 and HPV 16. Thirteen of 16 (81.3% sensitivity) biopsy samples confirmed to be CIN 2+ were positive for one of the five types presented in the PreTect HPV-Proofer assay. E6/E7 mRNA of one or more of four additional types, HPV 35, 51, 52 and 58, was found in 11 cases. Seven of the eight CIN3 cases were mRNA positive for one of the five types presented in the PreTect HPV-Proofer assay, which gave a sensitivity of 87.5 and 94.8% in specificity (accuracy of 94.6%). The last CIN3 case was DNA 66 positive.

We found 3.7% PreTect HPV-Proofer positives in women without CIN, no positives in CIN1 and 81.3% in CIN2+. The most ideal combinations of HPV types detected by NASBA giving maximum sensitivity and specificity (with the nine types) for detection of CIN2+ are shown in Table 3A. Table 3B presents the five types included in the PreTect HPV-Proofer assay.

### Concordance between HPV DNA and mRNA methods

Among samples that were both HPV mRNA and DNA positive, a 73.0% concordance in typing results was obtained when the criterion was full identity of all detected types including multiple infections. In 10 cases, we observed a discrepancy between one or more HPV types (Table 4) and because of lack of sample material, these cases could not be re-analysed to resolve typing differences.

Compatible results between HPV mRNA and DNA tests (meaning at least one common type in a given sample between these tests) were found for 83.8% of the positive cases.

Figure 3 gives an overview of the accuracy of each of the methods in percentage, presented in an ROC diagram.

## Discussion

In this study, cervical samples from 343 women have been investigated in a low-income setting by different cytological and HPV molecular methods to give an overview over test characteristics and to seek an alternative to cytology. To our knowledge, this is the first study performed in Africa that has included HPV mRNA analyses and that involved full gold standard information. As all samples were tested with all methods including histology, this may also be one of the rare studies related to cervical pre-cancerous stages without the need for correction of verification bias.

The prevalence of CIN2+ (4.7%) was high but not exceptional compared with other African settings in the same geographical area: 5.3% in Kinshasa (DR Congo) and 5.6% in Brazzaville (R Congo) (Sangwa-Lugoma *et al*, 2006; Arbyn *et al*, 2008).

Four of the 16 (25.0%) CIN 2+ cases were diagnosed by taking punch biopsy samples standardised at the 12 o’clock position, which are in accordance with previous results (Sellors *et al*, 2005; Jeronimo and Schiffman, 2006). The high PPV in this study should be explained as the consequence of the high-risk population that participated in the study and the sensitive verification process involving biopsy taking even in the absence of colposcopically suspicious areas. The inaccuracy of colposcopy was evident, most likely on the basis of only a single biopsy taken in most cases (Gage *et al*, 2006) and not merely because of a 2-week introduction course. The unsatisfactory rate for colposcopy was high (21%). A reasonable explanation based on age or other factors was not found.

By both PAP and LBC, a high proportion of histologically confirmed CIN2+ lesions were diagnosed as normal or low grade (10 of 16).

The DNA assay (Table 2), focusing on the 14 HR types, HPV 16 showed the highest prevalence for CIN2+ with a sensitivity of 31.3% and a specificity of 99.0%. The second most prevalent HPV type in CIN2+ was HPV 33. A combination of HPV 16 and 33 resulted in a sensitivity of 50.0% and a specificity of 98.7%. Hundred percent sensitivity could be achieved by including an octet of HPV16-33-18-51-66-56-45-35. Adding more types resulted in an increasing loss of specificity, as shown earlier by Schiffman *et al* (2005a).

An assay with 14 high-risk types yielded a specificity of 85.9%, which is relatively low compared with, for example, cytology that reached a specificity of 96.2%. A cocktail of HPV16-18-31-33 and 45 (similar to PreTect HPV-Proofer) in a DNA assay would give a sensitivity of 75.0% and a specificity of 94.6%. This is lower than those of PreTect HPV-Proofer assay (81.3 and 96.6%, respectively) for the same HPV types. On the other hand, DNA analysis with a different set of five types (i.e., HPV 16-33-18-51-66) yielded similar sensitivity and specificity values for CIN2+ (81.3 and 97.0%, respectively) as PreTect HPV-Proofer. The *P*-value for McNemar's *χ*^2^ test was 0.32 and 0.16 for differences in sensitivity and specificity, respectively, between DNA and mRNA testing when including the same number of HPV types.

For the mRNA-based assay, HPV 16 would also be the first type to be included (Table 3A) in an ideal assay and this would give a sensitivity similar to DNA detection and a slightly higher specificity (100.0 *vs* 99.0%). The second most prevalent mRNA HPV type in CIN2+ was HPV 18 and a combination of only HPV 16 and 18 showed a sensitivity of 56.3% and a specificity of 99.0%. Maximum sensitivity (93.8%) was attained by targeting six types: HPV 16-18-33-35-51-45, showing a specificity of 95.3%.

PreTect HPV-Proofer showed higher sensitivity for CIN2+ than for LBC or PAP, considering ASCUS+ as cutoff. Adding four more HPV mRNA types (HPV 35-51-52-58) resulted in the detection of two more CIN2+ cases. However, by including these four additional types, the number of false positives was increased by eight cases.

Finally, both the the PreTect HPV-Proofer (95% CI: 93.9–98.4 *vs* 81.4–89.6% *P*<0.0001) and the NASBA 9 types (95% CI: 90.6–96.4 *vs* 81.4–89.6% *P*<0.0001) were significantly more specific for CIN2+ than for HR-HPV DNA (14 types) detection.

The difference in specificity for detection of CIN2+ using five HPV mRNA types *vs* nine HPV mRNA types (96.6 *vs* 93.9%, respectively) was not significant. Nevertheless, the 2.5% higher accuracy obtained by PreTect HPV-Proofer and the favourable effects of using five types instead of nine types regarding cost and the follow-up in a low-resource setting give basis to prefer PreTect HPV-Proofer over a nine mRNA-type assay. The reason for including additional mRNA types was to determine whether one or more of these extra types could be particularly prevalent in this area in which no former HPV epidemiological data exist. The data did not give reason to believe that this was the case.

Small differences in HPV-type distribution in cervical carcinomas exist between different parts of the world. However, in the meta-analyses performed by Clifford *et al* (2003b), it is indicated that the five types, HPV 16, 18, 31, 33 and 45, are the most predominant HPV types detected in high-grade cervical neoplasia and comprise almost 97% of the HPV DNA types detected in cervical carcinomas in Europe and Northern America. The number is compensated for multiple infections (Clifford *et al*, 2003b, 2005). There are also strong indications that the same five types are the most predominant ones in cervical carcinomas in the rest of the world (Munoz *et al*, 2004; Smith *et al*, 2007). In a previous study, mRNA transcripts from these five types were detected in 89% of HPV-positive biopsies of cervical squamous cell carcinomas (88% by type-specific PCR) (Kraus *et al*, 2006).

The main problem with HPV DNA testing for all 14 HR types is the high test-positivity rate, namely, 18.5% in the population examined in this study.

This translates into twice the test positivity rate compared with the PreTect HPV-Proofer assay. Despite a very high negative predicted value of this assay (i.e., very few women negative with the HR HPV DNA (14 types) test will develop cancer), more than 75% of all HR HPV infections will not persist (Cuschieri *et al*, 2004, 2005) and therefore do not give rise to CIN2+, which results in a rather low PPV compared with, for example, PreTect HPV-Proofer (26.7 *vs* 56.5%).

Owing to the fact that a number of CIN2 or worse lesions spontaneously regress and that the golden standard, histology, often does not accurately identify the malignant disease, the ‘perfect test’ may therefore actually be located to the left side and below the 100% mark of sensitivity in the ROC diagram (Figure 3). The accuracy of PAP was calculated to be just below PreTect HPV-Proofer and LBC. The HR-HPV DNA-type detection assay is located quite far from PreTect HPV-Proofer, measured along the X-axis (1 specificity). In view of the DNA assay, a number of false positives exceeding the cytology test result accuracy may be regarded as not feasible for the clinical management system in low-income countries, where triage options are unavailable. It has been shown that women who are both positive with HPV DNA and mRNA are significantly more likely to harbour persistent infection compared with those in whom only DNA is detected at baseline (Kraus *et al*, 2006).

In many settings in low-income countries, there is a need for robust testing systems with good (but not necessarily the highest possible) sensitivity and specificity and applicable on the spot (giving the test result on the same day). A test targeting only the five most prevalent HPV types in cervical cancer could fulfil this criterion. In our study, such a five-type DNA test resulted in a sensitivity of 75.0% and a specificity of 94.6%, using CIN2+ as threshold for disease. With PreTect HPV-Proofer, the sensitivity and specificity were 81.3 and 96.6%, respectively. A reasonable hypothesis is that 18.7% of the CIN2 or worse lesions would have a low chance of progression. However, the cross-sectional design does not allow the confirmation of this hypothesis. A combination of both types of restriction and the fact that PreTect HPV-Proofer detects mRNA have resulted in a higher accuracy for PreTect HPV-Proofer compared with HR-HPV DNA. Even if the number of CIN2+ cases is relatively small in this study, we still see a contribution to the increasing sensitivity (75.0%–81.3) and specificity (94.6–96.6%) when including the same five HR HPV types in an assay performing an mRNA compared with a DNA-based assay.

The HPV DNA testing in Africa has been suggested by several experts, as has HPV mRNA testing. However, to date, there is limited information on the possibilities of using HPV-based tests in Africa. This study demonstrated for the first time that collection and storage of mRNA for molecular analysis is feasible in a low-income setting.

The most realistic way to screen for management of cervical cancer in low-income settings is by adopting a ‘screen-and-treat’ strategy (Denny *et al*, 2000, 2005). Therefore, the most desirable test should detect as few false positives and as many true-positive pre-cancer and cancer samples as possible (a high specificity in combination with a high PPV) to minimise both over- and undertreatment. This will make the test more useful in low-income countries, where health resources are extremely limited. Detecting mRNA as opposed to DNA has the potential of giving much more information about the activity of the oncogenes that are important for the development of cervical cancer. This in turn can give a higher clinical specificity, still with the same analytical sensitivity as PCR. The introduction of mRNA testing with a limited but area-specific set of HPV types in Africa (and low-income settings in general) could be a possibility in the near future because of the development of several sophisticated instruments (e.g., small battery-operated fluorescense readers) and also because of improvements in the storage of kit ingredients (e.g., primers and probes) at room temperature. The results from this study will be important for investigators in this field to optimise tests for use in low-income settings in general and in this area in particular.

Finally, HPV DNA tests specifically designed for use in low-resource areas have been developed. The *care*HPV test, based on the hybrid capture 2 (hc2) test (Qiagen, Gaithersburg, MD, USA), detecting 14 high-risk types, is one of these tests. *care*HPV is more feasible for usage in low-resource areas than is hc2; it is less expensive but is relatively nonspecific (Qiao *et al*, 2008).

Several companies, including NorChip AS, have developed Lab-on-a-chip technology (Zhang and Xing, 2007). This technology has already been adapted for performing hands-free Nucleic Acid Diagnostic (NAD) (Gulliksen *et al*, 2005), including complete extraction of RNA and DNA (Baier *et al*, 2009). The disposable chips are designed to include all reagents needed for complete NAD, including the detection of different mRNA targets, and may be mass produced to a low cost adapted to a low-resource market. The PreTect HPV-Proofer technology can be included with a number of types, adapted specifically for a certain region according to decisions made by the local health authorities.

### Strengths and weaknesses

Sample enrolment was performed strictly according to protocol, thus providing a high-quality data set and making extrapolations redundant. The first author was present during all sample enrolments to assist in any possible way. The collection of a biopsy sample from each woman reduces verification bias, but because only a single biopsy sample was taken, bias is not totally removed. The reason for taking a single biopsy sample was to minimise the possible discomfort and risk of infection for the women.

The cross-sectional study design does not allow for the possible detection of progression of pre-cancerous cases but because of ethical issues, we could not postpone the treatment, especially as this study was conducted in a low-resource setting in which difficulties in recalling the women are usually much higher and loss in follow-up is expected to be high.

Detectable infections observed during each of the gynaecological examinations were treated, if possible, free of charge. All women treated for CIN2 or worse infections were also asked to return to observe the treatment effect. All mRNA-positive but histology benign or low-grade women also had an opportunity to return for follow-up examinations, also free of charge. These results were not included in the study.

The HPV examination was carried out with a broad spectrum of methods to identify any possible HPV types. The examination of conventional PAP smears was performed by laboratories in Scandinavia and not by additionally including the local pathologist in the reviewing process because of logistic problems with sending smears between Scandinavia and Congo.

We cannot confirm HPV epidemiological data from this specific region, as the number of women included in the study is relatively small.

## Conclusions

This study shows that testing with nucleic acid-based assays is more sensitive than with either PAP or LBC. Only a few HPV types are needed in an assay to obtain a good sensitivity while maintaining a high specificity using CIN2+ as end point.

The combination of over two times higher PPV and a significantly higher specificity than HR-HPV DNA, in addition to a substantially higher sensitivity than cytological PAP at cutoff ASCUS+ (81.3 *vs* 66.7%), indicates a potential value of PreTect HPV-Proofer as a diagnostic method in areas without cytology-based screening possibilities.

In this study, PreTect HPV-Proofer showed a lower sensitivity for identifying CIN2+ compared with HR-HPV DNA typing with 14 types. Longitudinal studies are necessary to answer whether this difference in sensitivity is significant in terms of missed cancers. However, the pooled analyses indicate that the five types included in PreTect HPV-Proofer cover approximately 90% of HR HPV-positive cervical cancers worldwide (Munoz *et al*, 2004).

## Figures and Tables

**Table 1:** Test characteristics of the assays

			**Unsatisfactory samples**		**Accuracy for CIN2+**							
**Test method**	**Positive criterion**	**Test positivity rate** ***N* (%)**	**D+**	**D−**	**True positive**	**False negative**	**False positive**	**True negative**	**Sensitivity**	**Specificity**	**Positive predictive value**	**Negative predictive value**
PAP	⩾ASCUS	21/305 (6.9)	1	7	10	5	11	279	0.667	0.962	0.476	0.982
	⩾LSIL	16/305 (5.2)	1	7	9	6	7	283	0.600	0.976	0.563	0.979
	⩾HSIL	6/305 (2.0)	1	7	5	10	1	289	0.333	0.997	0.833	0.967
LBC	⩾ASCUS	20/301 (6.6)	1	11	11	4	9	277	0.733	0.969	0.550	0.986
	⩾LSIL	16/301 (5.3)	1	11	9	6	7	279	0.600	0.976	0.563	0.979
	⩾HSIL	7/301 (2.3)	1	11	5	10	2	284	0.333	0.993	0.714	0.966
Colposcopy	⩾Abnormal	103/252 (40.9)	2	59	11	3	92	146	0.786	0.613	0.107	0.980
	⩾High-grade	16/252 (6.3)	2	59	4	10	12	226	0.286	0.950	0.250	0.958
HR-HPV DNA (14 types)	1 of 14 types +	58/313 (18.5)	0	0	16	0	42	255	1.000	0.859	0.276	1.000
NASBA mRNA (9 types)	1 of 9 types +	33/313 (10.5)	0	0	15	1	18	279	0.938	0.939	0.455	0.996
PreTect HPV-Proofer	1 of 5 types +	23/313 (7.3)	0	0	13	3	10	287	0.813	0.966	0.565	0.990

The column ‘Positive criterion’ specifies the different criteria used within each method; ‘Test positivity rate’ gives the number of positive of total number of samples given each specified criterion for each method; The column ‘Unsatisfactory samples’ specifies number of unsatisfactory samples for each method with a CIN2+ (D+) and without a CIN2+ (D−) diagnosis. Unsatisfactory samples have been excluded from the accuracy calculations for detection of CIN2+.

**Table 2:** The most ideal combination of HPV DNA types giving maximum sensitivity and specificity for detection of CIN2+ for 14 HR-HPV DNA types

**HPV type in assay**	**# types**	**TP**	**FN**	**FP**	**TN**	**Sensitivity**	**Specificity**
0	0	0	16	0	297	0.000	1.000
HPV16	1	5	11	3	294	0.313	0.990
HPV16-33	2	8	8	4	293	0.500	0.987
HPV16-33-18	3	11	5	6	291	0.688	0.980
HPV16-33-18-51	4	12	4	6	291	0.750	0.980
HPV16-33-18-51-66	5	13	3	9	288	0.813	0.970
HPV16-33-18-51-66-56	6	14	2	12	285	0.875	0.960
HPV16-33-18-51-66-56-45	7	15	1	19	278	0.938	0.936
HPV16-33-18-51-66-56-45-35	8	16	0	27	270	1.000	0.909
HPV16-33-18-51-66-56-45-35-58	9	16	0	28	269	1.000	0.906
HPV16-33-18-51-66-56-45-35-58-68	10	16	0	29	268	1.000	0.902
HPV16-33-18-51-66-56-45-35-58-68-31	11	16	0	31	266	1.000	0.896
HPV16-33-18-51-66-56-45-35-58-68-31-39	12	16	0	33	264	1.000	0.889
HPV16-33-18-51-66-56-45-35-58-68-31-39-52	13	16	0	37	260	1.000	0.875
HPV16-33-18-51-66-56-45-35-58-68-31-39-52-59	14	16	0	42	255	1.000	0.859

Abbreviations: TP=true positive; FN=false negative; FP=false positive; TN=true negative.

**Table 3:** The most ideal combination of HPV mRNA types giving maximum sensitivity and specificity for detection of CIN2+, for the NASBA mRNA types (A) and the PreTect HPV-Proofer types, respectively (B)

**HPV type in assay**	**# types**	**TP**	**FN**	**FP**	**TN**	**Sensitivity**	**Specificity**
*(A) NASBA mRNA (nine types)*							
0	0	0	16	0	297	0.000	1.000
RNA16	1	5	11	0	297	0.313	1.000
RNA16-18	2	9	7	3	294	0.563	0.990
RNA16-18-33	3	12	4	3	294	0.750	0.990
RNA16-18-33-35	4	13	3	5	292	0.813	0.983
RNA16-18-33-35-51	5	14	2	7	290	0.875	0.976
RNA16-18-33-35-51-45	6	15	1	14	283	0.938	0.953
RNA16-18-33-35-51-45-31	7	15	1	14	283	0.938	0.953
RNA16-18-33-35-51-45-31-52	8	15	1	15	282	0.938	0.949
RNA16-18-33-35-51-45-31-52-58	9	15	1	18	279	0.938	0.939
							
*(B) PreTect HPV-Proofer (five types)*							
0	0	0	16	0	297	0.000	1.000
RNA16	1	5	11	0	297	0.313	1.000
RNA16-18	2	9	7	3	294	0.563	0.990
RNA16-18-33	3	12	4	3	294	0.750	0.990
RNA16-18-33-45	4	13	3	10	287	0.813	0.966
RNA16-18-33-45-31	5	13	3	10	287	0.813	0.966

Abbreviations: TP=true positive; FN=false negative; FP=false positive; TN=true negative.

**Table 4:** Discrepancy between DNA and mRNA testing

**Case**	**Histological end point**	**DNA result**	**RNA result**
1	⩾CIN2+	56	18
2	⩾CIN2+	16-30-35	16-18
3	⩾CIN2+	33-56	33-45
4	<CIN2+	72	18
5	<CIN2+	59	45
6	<CIN2+	31-82	51
7	<CIN2+	52	51-52
8	<CIN2+	31-66-42	31-58
9	<CIN2+	X (HR-EIA)	58
10	<CIN2+	35	58
